# Skin tone representation in dermatologic direct-to-consumer advertisements: a cross-sectional analysis and call to action

**DOI:** 10.1097/JW9.0000000000000101

**Published:** 2023-08-21

**Authors:** Chidubem A. V. Okeke, Joseph Tran, Ixavion Wright, Ginette A. Okoye, Cheryl M. Burgess, Angel S. Byrd

**Affiliations:** a Department of Dermatology, Howard University College of Medicine, Washington, DC; b Howard University College of Medicine, Washington, DC; c Center for Dermatology and Dermatologic Surgery, Washington, DC

**Keywords:** advertising, dermatology, media, skin of color

## Abstract

**Background:**

Direct-to-consumer advertisements (DTCAs) in medical marketing serve as a prominent modality to deliver information to an increasingly diverse audience of consumers and increase prescription sales. In dermatology, advertisements have the potential to shape the public’s opinions, aid in the understanding of skin conditions, and raise awareness of available treatments.

**Objective:**

To investigate and characterize the representation of skin tones in DTCAs.

**Methods:**

Nielsen ratings were utilized to identify the networks most watched by Black viewers in 2022. Programming on NBCUniversal, ABC, CBS, and FOX that aired in the District of Columbia, suburban Maryland, and Northern Virginia from June 2022 to July 2022 was reviewed for DTCAs. DTCAs were then analyzed to determine the skin tones of models and skin conditions depicted on models with darkly pigmented skin.

**Results:**

Of the 106 DTCAs related to dermatologic conditions, there were 13 unique advertisements featuring 32 unique models. Four advertisements depicted the skin condition on darkly pigmented skin tones. Using the Monk Skin Tone (MST) scale to assess the 32 unique individuals, only 25% (*n* = 8) were rated at an MST 7 or above, and 6.25% (*n* = 2) were rated at an MST 10.

**Limitations:**

This study has the limitation of only sampling DTCAs from Washington, District of Columbia which does not fully represent all dermatology-related DTCAs in the United States.

**Conclusion:**

Results of this content analysis demonstrate that the number of persons of color within dermatologic DTCAs is 23%, whereas there are 13.6% Black individuals in the 2021 US census. This suggests that DTCAs are becoming more diverse since 2018. However, findings also show that the vast majority of DTCAs do not include models with darkly pigmented skin, and there remains a lack of advertisements depicting skin disease among people of color. Given the role of DTCAs in informing and aiding patients’ requests for prescription drugs, representation of all skin tones is essential for this communication to be effective, especially in the field of dermatology.

**What is known about this subject in regard to women and their families?**

- The promotion of prescription drugs is aimed at the general public, which includes adult women, adult men, teenagers, and children.
- Exposure to direct-to-consumer advertisements (DTCAs) leads to increased searching for information and promotion of newly approved therapies.
- While rates of dermatologic conditions such as psoriasis may be higher in non-Latino white populations, they are not rare among people of color (POC), and POCs are less likely to seek dermatologic care for them.
- Therefore, DTCAs may play a role in misperceptions by darkly pigmented POC that they are not at risk for certain skin conditions.

**What is new from this article as messages for women and their families?**

- There is a decreased number of models with darkly pigmented skin in DTCAs for conditions that affect people of color.

## Background

Direct-to-consumer advertisements (DTCAs) deliver information and promote the sale of prescription and over-the-counter (OTC) drugs to an increasingly diverse audience of consumers.^1^ In the United States, approximately $9.6 billion is spent on DTCAs each year, and DTCAs related to dermatology account for $605 million.^1^ Yet, research demonstrates that people of color (POC) are continuously underrepresented or misrepresented in these advertisements.^2,3^ In 2019, Holmes et al.^2^ found that in psoriasis DTCAs, only 6.2% of the characters portrayed were Black, whereas 92.6% of the characters were White.^2^ While rates of dermatologic conditions such as psoriasis may be higher in non-Latino White populations, they are not rare among POC, and POC are less likely to seek dermatologic care for them. Therefore, DTCAs may play a role in misperceptions by darkly pigmented POC that they are not at risk for certain skin conditions. The aim of this study was to examine the content of dermatologic DTCAs to assess whether there is an equitable inclusion of POC with darker skin tones.

## Methods

**Fig. 1.**
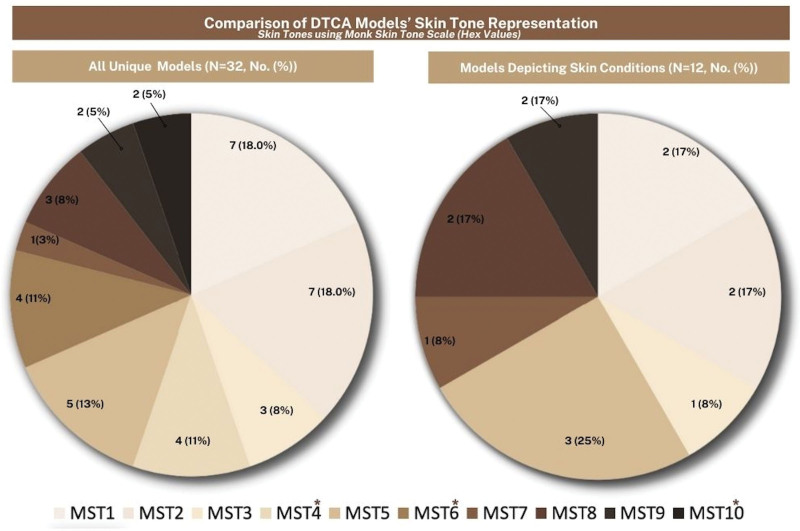
Flow diagram according to Strengthening the Reporting of Observational Studies in Epidemiology (STROBE) guidelines.^5^

We analyzed 657 DTCAs aired between June 2022 and July 2022 during the hours of 5 PM Eastern Standard Time to 11 PM Eastern Standard Time. Five local networks (NBCUniversal, ABC, CBS, WUSA9, and FOX), aired in the District of Columbia, suburban Maryland, and Northern Virginia, were chosen based on Nielsen’s Top 10 prime broadcast programs among African Americans.^4^ The premise for this selection was that these networks were likely to be watched by Black viewers, and branded commercials may contain more diverse individuals. Infomercials and promotional commercials were excluded from the analysis. The remaining advertisements were sorted between dermatologic prescriptions and OTC DTCAs. Only nonduplicate commercials were used to assess the content. In the determination of unique advertisements, 15-second spotlight DTCAs (same content but a shorter version of a full commercial) were not considered unique. A flow diagram of this process is presented in Fig. 1.

### Coding and analysis

**Fig. 2.**
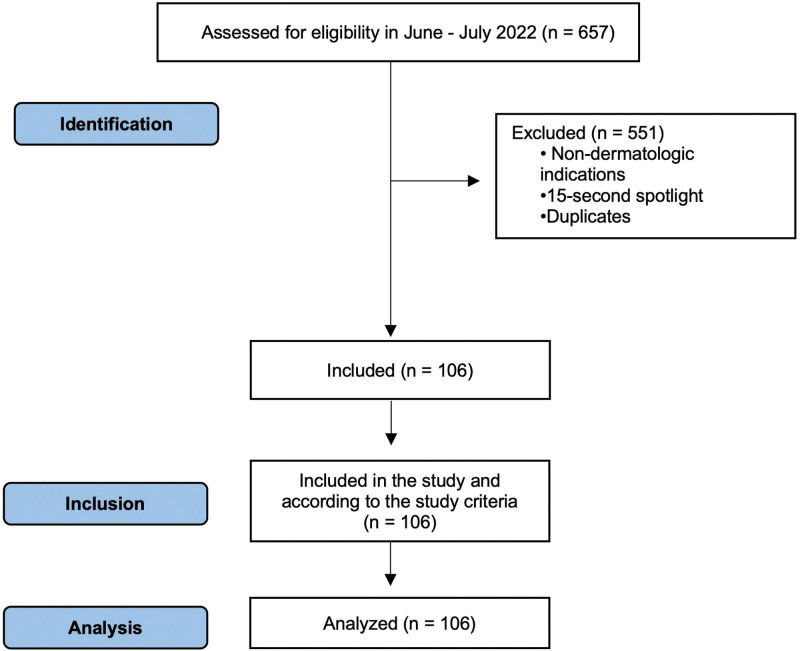
Comparison of DTCA Models’ Skin Tone representation. *, Skin tones not represented in models (MST4, MST6, and MST10). DTCA, direct-to-consumer advertisements.

Research assistants (JT and IW) independently viewed and coded DTCAs 3 separate times. In each viewing, the skin tone for each model was determined using a 10-point &Monk Skin Tone (MST) Scale^6^ (Fig. 2). The MST Scale is a 10-shade scale designed to represent a broader range of skin tones. All coding discrepancies were resolved by a third-party independent researcher (ET) who conducted a final review of the data. All calculations for the analysis were performed using the Microsoft Excel 2007 software package (Microsoft Corporation, Redmond, WA, USA).

## Results

### Sample characteristics

Of the 657 DTCAs, 16.1% (106/657) were devoted to skin conditions. There were 13 unique advertisements that represented 11 products and 5 different skin conditions (Table 1). There were 10 DTCAs for prescription medications and 3 for dermatological OTC products. The persons portrayed in these advertisements were both actors and real patients (Table 1), for ease, we will refer to each as a model. Of the dermatologic DTCAs, there were 32 unique individuals depicted, and 53.12% (*n* = 17) were perceived to be models of color. Additionally, 4 POC models depicted the advertisement’s skin condition (wrinkle development [*n* = 1], pruritus [*n* = 1], mild to moderate atopic dermatitis [*n* = 1], and moderate to severe plaque psoriasis [*n* = 1]). The other 13 models did not depict skin conditions, and their roles were either minor or noncontributory to the advertisement. The redness associated with pruritic skin was digitally enhanced to appear more pronounced on a darkly pigmented model. In addition, 25% of models (*n* = 8) were rated at MST 7 or above, of which 2 (6.25%) were rated at MST 10 (Fig. 2).

**Table 1:** Dermatology-DTCA product types and frequency

Product type	Frequency played	Indication	Actual patients (Y/N)
Total advertisements			
Over-the-counter	5	N/A	N/A
Prescription	101	N/A	N/A
Over-the-counter			
Adapalene gel 0.1%	2^a^	Acne	N
Hydrocortisone 1% topical cream	3	Anti-itch	N
Prescription (generic name)			
Secukinumab	2	Moderate to severe plaque psoriasis	Y
OnabotulinumtoxinA	3	Rhytides	Y
Apremilast	4	Mild, moderate, and severe plaque psoriasis	N
Dupilumab	6	Moderate to severe atopic dermatitis	N
Ruxolitinib	11	Mild to moderate atopic dermatitis	N
Risankizumab	14	Moderate to severe plaque psoriasis	N
Upadacitinib	19	Moderate to severe atopic dermatitis	N
Abrocitinib	35	Moderate to severe atopic dermatitis	N

DTCA, direct-to-consumer advertisements.

a Two unique products.

## Discussion

Although this content analysis revealed that POC inclusion is greater than that observed in the Holmes et al.^2^ analysis of psoriasis and eczema DTCAs, our findings highlight that further efforts are warranted to increase the skin tone representation of POC portrayed in DTCAS. Due to the subjective limitations of Fitzpatrick Skin Type (FST) for minimal erythema dose criteria, the MST scale was used to evaluate and visualize the diverse range of skin tones in DTCAs. Multiple studies have shown that while self-reported FST is useful in identifying burn risk from UV damage in tones (FST I-III) it is less efficient and accurate in nonwhite tones (FST IV-VI).^7–10^ The FST is not an appropriate substitute for skin color, race, or ethnicity, though it is often incorrectly used as such.^11^ One study showed that FST is often conflated with race/ethnicity, showing that 31% of surveyed dermatologists utilized FST to characterize race, 47% to describe skin color, and 22% do both, which lies outside the original scope of the scale’s use.^7^ As such, the MST scale was utilized since an unpublished study showed that participants viewed the scale to be more inclusive and representative of their skin tone when compared to FST. Study participants perceived the MST scale to be as representative as a 40-point skin tone palette by a makeup brand.^12^ The use of the MST scale more accurately showed the disparity in skin color representation in the advertisements and, as previous studies have mentioned, may point to a need for updated methods of measuring and representing skin color, race, and ethnicity for different research purposes.^7,13^ Ultimately, increasing the portrayal of skin conditions on all skin tones may aid in reducing the disparities in outcomes seen in communities of color by increasing health communication to the public.

Herein, models with lighter pigmented skin (MST 6 or below) predominated DTCAs. Another notable finding is that there is a lack of accurate depictions of atopic dermatitis, psoriasis, and acne in darkly pigmented individuals. For example, an OTC anti-itch product utilized an animation to show red, inflamed skin on a darkly pigmented person of color. This portrayal is realistic in lighter complexions, but inaccurate in darker complexions. In darker skin tones, redness may appear darker brown, purple, or gray in color and may be missed by evaluating clinicians.^14^ Advertisements that include these nuances may be more effective in helping darkly pigmented patients identify dermatologic diseases.

The social identity theory is a concept that explains how individual behaviors are influenced by interactions with others.^15^ Hence, this self-identification directly affects a consumer’s perception and ultimately their opinion. Many studies highlight this framework to support the effectiveness of DTCAs in health communication.^3,16,17^ Thus, the lack of diverse representation of individuals with darkly pigmented skin may lead to the failure of patients to self-identify with the models portrayed, which severely limits the effectiveness of DTCAs as a means of health communication with the general public.

For example, African Americans are at a disproportionate risk of developing certain skin conditions, such as atopic dermatitis.^18^ The lack of diverse visual representation in DTCAs aired during programming targeted toward African Americans^4^ may discourage this population from seeking available treatments. As the US population continues to diversify, it’s important to recognize that representation of various skin tones should not be limited. DTCAs help patients of all backgrounds gain greater awareness of available treatments.^19^ Therefore, it is necessary that DTCAs include a diverse range of individuals to better engage the present-day patient. Hopefully, these efforts will increase dermatologists’ awareness of these advertisements and their potential influence on patients.

Ultimately, increasing the portrayal of skin conditions on all skin tones may aid in reducing the disparities in health outcomes prevalent in communities of color. The United Nations International Children’s Emergency Fund (UNICEF) has a guide^20^ for ensuring diversity in programming for diverse audiences, which should be considered when marketing and advertising dermatology products. Championing diversity, equity, justice, and inclusion across all levels, including, but not limited to, the workforce, product development, and marketing will promote change.

## Limitations

This study has the limitation of only sampling DTCAs from Washington, District of Columbia which does not fully represent all dermatology-related DTCAs in the United States. Our time frame of 2 months also limits the sample size of DTCAs analyzed in this work. Finally, we also face the challenge of inherent bias associated with the investigator categorization of skin tones.

## Conclusion

This content analysis revealed 2 major realizations: (1) there remains a disproportionate amount of people of color depicting dermatological conditions and (2) of those represented, models with lighter pigmented skin predominated while a small number had darker pigmented skin (MST 7 or above). Although this study is limited in its ability to examine the effect of DTCAs on patients, it supports the need for research exploring the impact of DTCAs on skin of color patients’ understanding of common skin conditions and therapies in efforts to improve equality and health equity outcomes.

## Conflicts of interest

None.

## Funding

None.

## Study approval

N/A.

## Author contributions

CAVO and ASB have full access to all the data in the study and take responsibility for the integrity of the data and the accuracy of the data analysis. Study concept and design: CAVO. Acquisition, analysis, or interpretation of data: CAVO, JT, IW. Drafting of the manuscript: CAVO, JT, IW. Critical revision of the manuscript for important intellectual content: All authors.

## Acknowledgments

CAVO is the inaugural recipient of the Women’s Dermatologic Society-La Roche Posay Dermatology Fellowship. ASB is the inaugural recipient of the Skin of Color Society Career Development Award as well as the Society for Investigative Dermatology Freinkel Diversity Fellowship Award, and recipient of the Robert A. Winn Diversity in Clinical Trials Development Award, funded by Bristol Myers Squibb Foundation. The authors would like to acknowledge Elizabeth Tran (University of Maryland) for her contributions.
